# In Situ Nitrogen Functionalization of 2D-Ti_3_C_2_T*_x_*-MXenes for High-Performance Zn-Ion Supercapacitor

**DOI:** 10.3390/molecules27217446

**Published:** 2022-11-02

**Authors:** Abdul Mateen, Mohd Zahid Ansari, Qasim Abbas, Ahmed Muneeb, Ahmad Hussain, Elsayed tag Eldin, Fatimah Mohammed Alzahrani, Norah Salem Alsaiari, Shafaqat Ali, Muhammad Sufyan Javed

**Affiliations:** 1 Department of Physics, Beijing Key Laboratory of Energy Conversion and Storage Materials, Beijing Normal University, Beijing 100084, China; 2 School of Materials Science and Engineering, Yeungnam University, Gyeongsan 712749, Korea; 3 Department of Intelligent Manufacturing, Yibin University, Yibin 644000, China; 4 Department of Botany, Division of Science and Technology, University of Education, Lahore 54000, Pakistan; 5 Department of Physics, The University of Lahore Sargodha campus, 40100 Sargodha, Pakistan; 6 Faculty of Engineering and Technology, Future University in Egypt, New Cairo 11835, Egypt; 7 Department of Chemistry, College of Science, Princess Nourah bint Abdulrahman University, Riyadh 11671, Saudi Arabia; 8 Department of Biological Sciences and Technology, China Medical University, Taichung 40402, Taiwan; 9 Department of Environmental Sciences, Government College University, Faisalabad 38000, Pakistan; 10 School of Physical Science and Technology, Lanzhou University, Lanzhou 730000, China

**Keywords:** MXene, nitrogen functionalization, specific capacitance, zinc ion supercapacitor

## Abstract

Zinc (Zn) ion supercapacitors (ZISCs) have attracted considerable attention as a viable energy storage technology because they are cost-effective, safe, and environmentally friendly. However, cathode materials with suitable properties are rare and need to be explored. In this regard, metal carbides (MXenes) are a good choice for capacitive energy storage, but they exhibit low capacitance. The energy storage performance of MXenes can be bossed using functionalization with heteroatom doping, e.g., nitrogen (N), to simultaneously modify ZISCs’ fundamental characteristics and electrochemical properties. Herein, we present an in-situ *N*-functionalization of Ti_3_C_2_T*_x_*-MXene via a hydrothermal reaction with urea (denoted as *N*-Ti_3_C_2_T*_x_*-MXene). *N*-functionalization into Ti_3_C_2_T*_x_*-MXene raised Ti_3_C_2_T*_x_*-MXene’s interlayer spacing and boosted the Zn-ion storage in 1 M ZnSO_4_ electrolyte. The *N*-Ti_3_C_2_T*_x_*-MXene electrode delivered an excellent specific capacitance of 582.96 F/g at 1 A/g and retained an outstanding cycle stability of 94.62% after 5000 cycles at 10 A/g, which is 1.8 times higher than pristine Ti_3_C_2_T*_x_*-MXene at identical conditions. Moreover, the *N*-Ti_3_C_2_T*_x_*-MXene//Zn device demonstrated a maximum capacitance of 153.55 F/g at 1 A/g, retained 92% of its initial value after 5000 cycles, and its Coulombic efficiency was ~100%. This strategy considerably reduced Ti_3_C_2_T*_x_*-MXene nanosheet restacking and aggregation and enhanced electrochemical performance. Further, this research elucidated *N*-Ti_3_C_2_T*_x_*-MXene’s charge–storage process and offered a fresh approach to the rational design of novel electrode materials for ZISCs.

## 1. Introduction

Expanding energy crises and environmental degradation have provided a powerful impetus for the development of safe, environmentally sustainable, and inexpensive energy storage technologies [1,2]. Further, humans’ extensive energy demands require large-scale research into electrochemical energy storage and conversion technologies, which can store energy more efficiently [3,4]. Recently a significant increase in research efforts directed toward discovering energy storage devices that are safe and affordable have been connected with high-performing energy conversion devices [5,6]. Particular consideration has been given to designing rechargeable batteries [7] and supercapacitors (SCs) [8]. SCs are simple and risk-free technologies, but their low energy density is a primary limitation. Lithium-ion batteries (LIBs) have been widely commercialized for electronic devices because of their low weight, excellent energy density, and outstanding performance [9]. However, scaling up LIBs is restricted by significant safety and environmental issues [10]. Aqueous Zn-ion storage has drawn much interest as a potentially useful aqueous electrolyte-based device for widespread energy storage, because of the Zn anode’s exceptional stability in aqueous electrolyte and ease of fabrication [11,12,13]. The research and development of zinc ion supercapacitors (ZISCs) with excellent performance is therefore of utmost importance.

Electrode material is essential in determining aqueous ZISCs’ electrochemical performance. There have been numerous efforts to investigate novel electrode materials for high-performance ZISCs, including organic compounds [14,15], Prussian blue [16], and transition metal oxides [17,18]. Designing aqueous ZISCs with suitable capacitance, extended cycle life, and remarkable rate performance remains challenging. MXenes are a novel class of two-dimensional (2D) materials that have applications in energy conversion and storage [19]. MXenes are typically 2D transition metal nitrides, carbides and/or carbonitrides with the general formula M_n+1_X_n_T*_x_*, where M denotes transition metal, n = 1–4, X denotes nitrogen and/or carbon, and T denotes different surface termination groups, such as –OH, –F or –O [20,21]. Owing to its metallic conductivity, layered structure, superior mechanical characteristics, and surface hydrophilicity [22], MXenes have already proven to be attractive options as electrode material in SCs, zinc ion batteries, ZISCs, and sensors [19,23,24]. Unfortunately, similar to many other 2D materials, MXenes have high interlayer restacking and aggregation, which reduces their electrochemical performance. In particular, MXenes with multi-layered accordion-like architectures exhibit exceptionally low water dispersibility [25]. Multi-layers can be separated into fewer further dispersible layers using ultrasonication. However, MXene layers quickly re-stack and agglomerate when sonication stops [26,27]. There have been several attempts to address these issues [7,28]; of these, nitrogen (*N*)-functionalization of MXene nanosheets is an excellent strategy to boost MXene’s performance. Additionally, to achieve excellent improvements in ZISCs’ performance, it is essential to incorporate pseudocapacitive properties into the electrode materials [29]. For instance, incorporating heteroatoms, such as N, into carbon-based materials has proven to be an efficient and successful strategy for improving their electrochemical characteristics [30]. *N*-functionalized carbon-based materials offer enhanced electrical conductivity and surface wettability and are employed as electrode materials for high-performance ZISCs [31]. 

This paper presents a straightforward approach for preparing a novel type of *N*-functionalized Ti_3_C_2_T*_x_*-MXene (*N*-Ti_3_C_2_T*_x_*-MXene) via a hydrothermal technique with urea; *N*-functionalization into Ti_3_C_2_T*_x_*-MXene raised interlayer spacing. The *N*-Ti_3_C_2_T*_x_*-MXene electrode demonstrated a maximum capacitance of 582.96 F/g at 1 A/g and retained 94.62% of its initial value for 5000 cycles at 10 A/g. In addition, the *N*-Ti_3_C_2_T*_x_*-MXene//Zn device demonstrated a maximum specific capacitance of 153.55 F/g at 1 A/g, retention of 92% for 5000 cycles, and Coulombic efficiency equal to ~100%. This strategy considerably reduces Ti_3_C_2_T*_x_*-MXene nanosheet restacking and aggregation and enhances electrochemical performance. Further, this research elucidated *N*-Ti_3_C_2_T*_x_*-MXene’s charge–storage process and offered a fresh approach to the rational design of novel electrode materials for ZISCs.

## 2. Experimental Method

### 2.1. Preparation of Ti_3_C_2_T_x_-MXene

To prepare layered Ti_3_C_2_T*_x_*-MXene, Ti_3_AlC_2_ MAX powder (1 g) was mixed in 20 mL of HF (50 wt.%) containing solution at room temperature (RT) and constantly agitated for 90 h. The resultant Ti_3_C_2_T*_x_*-MXene solution was rinsed with water and then centrifuged at 1500 rpm for 15 min to attain a pH of ~6. The Ti_3_C_2_T*_x_*-MXene solution was filtered using a polyvinylidene fluoride (PVDF) membrane. The obtained Ti_3_C_2_T*_x_*-MXene powder was vacuum-dried at RT.

### 2.2. Synthesis of N-Ti_3_C_2_T_x_-MXene

First, 1 g of Ti_3_C_2_T*_x_*-MXene was dissolved in 100 mL DI water. After that, urea was dropped into the Ti_3_C_2_T*_x_*-MXene solution, which was continuously agitated. The proportion of urea to Ti_3_C_2_T*_x_*-MXene by weight was 1:30. The mixture was placed in an autoclave. The solution of urea and Ti_3_C_2_T*_x_*-MXene underwent a hydrothermal reaction for 6 h at 160 °C. Next, the nitrogen-functionalized Ti_3_C_2_T*_x_*-MXene (*N*-Ti_3_C_2_T_x_-MXene) was synthesized by letting the autoclave temperature naturally fall to RT. Finally, the *N*-Ti_3_C_2_T*_x_*-MXene was cleaned using water before being dried at ambient temperature in a vacuum.

### 2.3. Physical Characterization 

The sample was structurally analyzed using X-ray diffraction (XRD) (X’Pert Pro PANalytical), with a Cu Kα radiation of 0.15406 nm wavelength λ. The prepared sample’s morphology was analyzed using a transmission electron microscope (TEM JEM-2100F, JEOL). The material’s chemical states were analyzed using Raman spectroscopy (HJY Lab RAM Aramics 70 France). Elemental composition was investigated using X-ray photoelectron spectroscopy (XPS). Experiments involving the adsorption and desorption of nitrogen were carried out using a Brunauer–Emmett–Teller (BET) analyzer to determine surface area and pore structure. 

### 2.4. Electrochemical Measurements 

For three electrode configurations, the working electrode consisted of 80% *N*-Ti_3_C_2_T*_x_*-MXene, 10% conductive carbon black, and 10% polyvinylidene fluoride (PVDF) as a binder. A homogenous slurry was prepared using *N*-Methyl-2-Pyrrolidone (NMP) as a solvent, which was pasted on a 1 × 1 cm^2^ piece of pre-treated carbon cloth (CC) before being dried in a vacuum oven at 60 °C for 6 h. A platinum plate, with Ag/AgCl as a reference electrode using 1 M ZnSO_4_ electrolyte, was employed as a counter. To fabricate aqueous ZISCs, the previous working electrode was served as positive, with Zn as negative, in 1 M ZnSO_4_. An electrochemical workstation (CHI 660E, Wuhan, China) was used to perform cyclic voltammogram (CV) and galvanostatic charge–discharge (GCD) tests, and take electrochemical impedance spectroscopy (EIS) measurements. 

### 2.5. Calculations

(1) $$C_{sp}=\frac{I\Delta t}{m\times \Delta V}$$

(2) $$C_{d}=\frac{I\Delta t_{d}}{M\times \left(V\right)}$$

(3) $$E=\frac{1}{2}C_{d}V^{2}\times \frac{1000}{3600}$$

(4) $$P=\frac{E}{\Delta t}$$

(5) $$\eta \;\left(\%\right)=\frac{\Delta t_{d}}{\Delta t_{c}}\times 100$$

where *C*_*d*_ and *C*_*sp*_ denote capacitance for two- and three-electrode configuration, respectively; *E* and *P* denote energy and power densities, respectively; *η* denotes Coulombic efficiency; *I*(A) denotes current; *M* denotes the mass of two-electrode configurations; Δ*t* (s) denotes discharge time; and *V*(V) denotes the potential window. 

## 3. Results and Discussion

**Figure 1:**
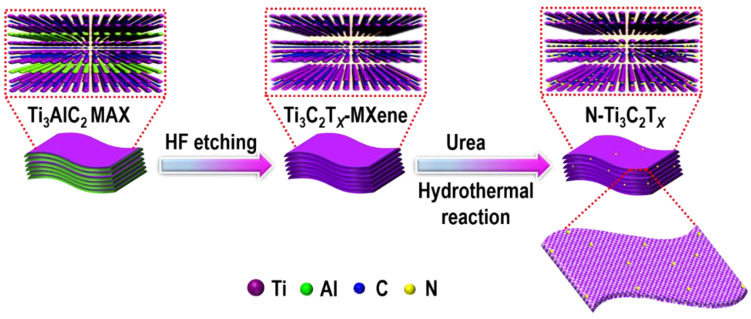
A schematic diagram of *N*-Ti_3_C_2_T*_x_*-MXene’s preparation.

Figure 1 illustrates the preparation method for *N*-functionalized Ti_3_C_2_T*_x_*-MXene (*N*-Ti_3_C_2_T*_x_*-MXene). First, multilayer Ti_3_C_2_T*_x_*-MXene with slightly increased layer spacing was formed via selective removal of the Al layer of Ti_3_AlC_2_ MAX using hydrofluoric acid (HF). During this procedure, several *O*-containing groups formed a negative charge on MXene nanosheets [32,33]. *N*-Ti_3_C_2_T*_x_*-MXene was subsequently fabricated using a hydrothermal process with urea. 

**Figure 2:**
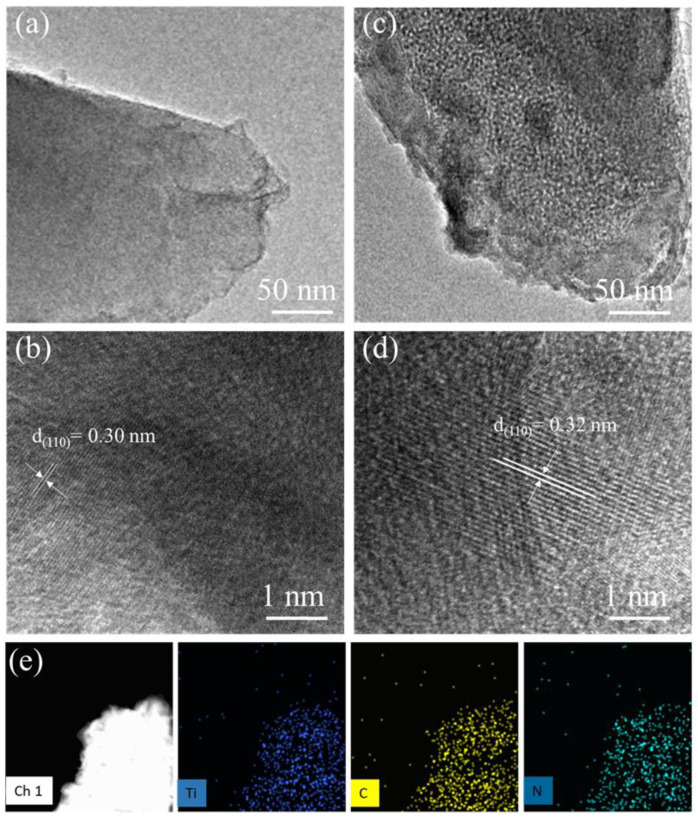
Low-resolution TEM images of (**a**) Ti_3_C_2_T*_x_*-MXene and (**b**) *N*-Ti_3_C_2_T*_x_*; high resolution TEM images of (**c**) Ti_3_C_2_T*_x_*-MXene and (**d**) *N*-Ti_3_C_2_T*_x_*; and (**e**) EDS elemental mapping.

A transmission electron microscope (TEM) was employed to analyze the micromorphology and microstructural development of both Ti_3_C_2_T*_x_*-MXene and *N*-Ti_3_C_2_T*_x_*-MXene. Figure 2a shows a low-resolution TEM image of pristine Ti_3_C_2_T*_x_*-MXene, which exhibits translucent and smooth nanosheets that ultimately overlapped to produce a wrinkled structure. Figure 2c is a TEM image of *N*-Ti_3_C_2_T*_x_*-MXene, which reveals the porous structure of *N*-functionalized MXene nanosheets. Figure 2b shows a high-resolution TEM image of Ti_3_C_2_T*_x_*-MXene, which exhibits a crystallite with an interplanar spacing of d_110_ = 0.30 nm, corresponding to the (110) plane. After *N*-functionalization, the interlayer spacing in the *N*-Ti_3_C_2_T*_x_*-MXene sample increased to d_110_ = 0.32 nm, as shown in Figure 2d. The increased interlayer spacing facilitated the interfacial charge transfer and electrolytic ion’s accessibility to electroactive areas. Moreover, the presence of large pores on *N*-Ti_3_C_2_T*_x_*-MXene nanosheets enhanced ion movement within the material, which enhanced its electrochemical performance. Figure 2e illustrates a uniform distribution of *N*, T, and C across the whole material via energy-dispersive X-ray spectroscopy (EDS) for *N*-Ti_3_C_2_T*_x_*-MXene. 

**Figure 3:**
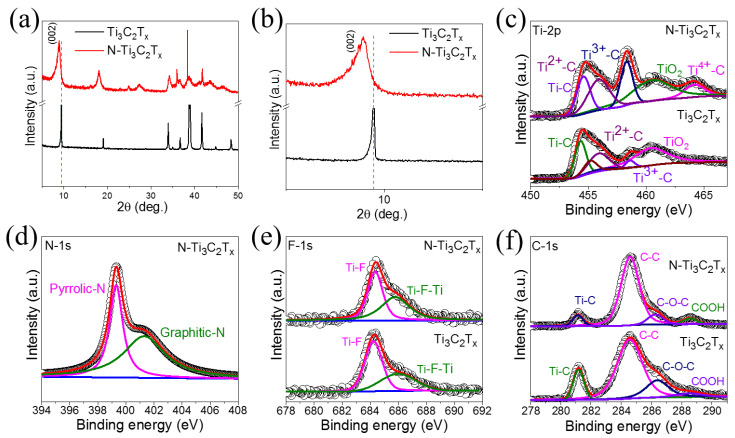
(**a**,**b**) XRD images of Ti_3_C_2_T*_x_*-MXene and *N*-Ti_3_C_2_T*_x_*; deconvoluted XPS spectra of (**c**) Ti-2p; (**d**) *N*-1s; (**e**) F-1s; and (**f**) C-1s.

Figure 3a,b depict X-ray diffraction (XRD) images of Ti_3_C_2_T*_x_*-MXene and *N*-Ti_3_C_2_T*_x_*-MXene. Ti_3_C_2_T*_x_*-MXene’s XRD pattern has a characteristic peak less than 10°, which confirms that Ti_3_AlC_2_ was successfully etched to fabricate Ti_3_C_2_T*_x_*-MXene [34]. XRD images of *N*-Ti_3_C_2_T*_x_*-MXene show that the *N*-functionalization approach had minimal effect on Ti_3_C_2_T*_x_*-MXene’s phase structure; the only notable change was that the (002) peak moved toward a lower diffraction angle associated with increased spacing. After further examination, it was discovered that the (002) peak was situated at approximately 9.3°, which was associated with a 0.96 nm interlayer spacing. Following *N*-functionalization, the position of the (002) peak changed to 8.9°, and the interlayer spacing was modified to 0.99 nm (Figure 3b). Increased interlayer spacing might have efficiently exploited the potential area for Zn^2+^ accommodation, implying a high capacitance. Further, Figure 3c shows high resolution Ti-2p XPS spectra, which were deconvoluted into five components: Ti-C (454.65 eV), Ti^2+^-C (456.05 eV), Ti^3+^-C (458.35 eV), TiO_2_ (460.74 eV), and Ti^4+^-C (464.21 eV). After *N*-functionalization, a new peak appeared at Ti^4+^-C (464.21 eV). Notably, each peak’s intensity was enhanced after *N*-functionalization, suggesting that Ti_3_C_2_T*_x_*-MXene underwent partial oxidation during the *N*-functionalization process. *N*-Ti_3_C_2_T*_x_*-MXene’s deconvoluted *N*-1s XPS spectra are shown in Figure 3d, which shows peaks at 399.30 eV corresponding to pyrrolic-N, whereas the peak at a 401.33 eV binding energy corresponds to graphitic-N. The contribution of pyrrolic-N and graphitic-N groups proved the improvement in the sample’s electrical conductivity and electrochemical activity. Figure 3e depicts the F-1s spectrum, which reveals peaks at 284.39 and 285.79 eV, related to Ti-F and Ti-F-Ti, respectively, indicating the presence of the -F group generated by HF etching. The deconvoluted C-1s spectrum is depicted (Figure 3f). This spectrum displays four peaks at binding energies 281.15, 284.61, 286.25, and 288.51 eV, related to the Ti–C, C–C, C–O–C, and COOH bonds, respectively. 

**Figure 4:**
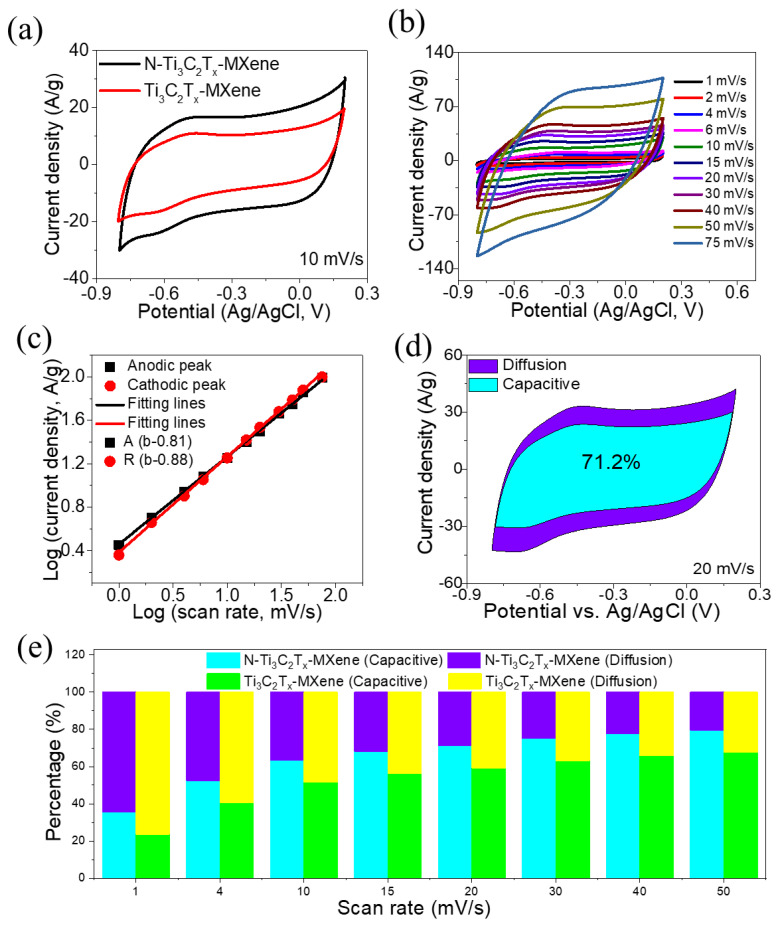
(**a**) CVs of Ti_3_C_2_T*_x_*-MXene and *N*-Ti_3_C_2_T*_x_*-MXene at -0.8-0.2 V; (**b**) CVs of *N*-Ti_3_C_2_T*_x_*-MXene at different sweep rates; (**c**) calculation of *b*-values log (current density) versus log (sweep rate); (**d**) percentage contribution of diffusion/capacitive mechanisms; and (**e**) percentage contribution of diffusion/capacitive mechanisms at different sweep rates (1–50 mV/s).

Using a three-electrode configuration in 1 M ZnSO_4_, the electrochemical performance of pristine Ti_3_C_2_T*_x_*-MXene and *N*-Ti_3_C_2_T*_x_*-MXene were investigated. Figure 4a displays the CVs of pristine Ti_3_C_2_T*_x_*-MXene and *N*-Ti_3_C_2_T*_x_*-MXene electrodes across the potential window range of −0.8–0.2 V. Both CV curves have a characteristic rectangular shape with anodic and cathodic peaks, suggesting that both electrodes retained their capacitive behavior. However, the individual capacitances displayed significant variation, as seen in the area under the CV curves. Compared to the pristine Ti_3_C_2_T*_x_*-MXene electrode, an enhanced specific capacitance can be inferred from the larger CV area for *N*-Ti_3_C_2_T*_x_*-MXene due to the outstanding conductivity and interconnectivity of *N*-Ti_3_C_2_T*_x_*-MXene nanosheets. Figure 4b depicts CVs of *N*-Ti_3_C_2_T*_x_*-MXene at various sweep rates (1–75 mV/s). Even when the sweep rate was very high (75 mV/s), *N*-Ti_3_C_2_T*_x_*-MXene displayed extremely capacitive behavior, excellent ion responsiveness, and good rate capabilities, with slight shifts in cathodic and anodic peaks [35]. Additionally, the mechanism for charge storage in electrodes was analyzed using a power law study of electrochemical kinetics [36].

*i*(*V*) = *a.v^b^* (6)

log (*i*) = b log (*v*) + log (*a*) (7)

where *v* denotes the sweep rate, *i* denotes the peak current density, and *a* and *b* denote arbitrary constants. A *b*-value = 0.5 implied that capacitance was regulated via ionic diffusion, whereas *b*-value = 1.0 showed that the capacitive mechanism dominated during charge–discharge. Figure 4c shows corresponding anodic and cathodic *b*-values of 0.81 and 0.88, respectively, demonstrating the synchronous diffusion and capacitive-controlled mechanisms in the electrochemical reaction of the *N*-Ti_3_C_2_T*_x_*-MXene. 

Figure 4d demonstrates that, at a sweep rate of 20 mV/s, *N*-Ti_3_C_2_T*_x_*-MXene stored charge 28.8% through a diffusion-controlled mechanism and 71.2% through a capacitive-controlled mechanism. Furthermore, Figure 4e illustrates the capacitive- and diffusion-controlled mechanisms for pristine Ti_3_C_2_T*_x_*-MXene and *N*-Ti_3_C_2_T*_x_*-MXene at various sweep rates (1 to 50 mV/s). The capacitive-controlled mechanism rose as the sweep rate increased, suggesting that the capacitive mechanism dominated the total capacitance, particularly at high sweep rates. 

**Figure 5:**
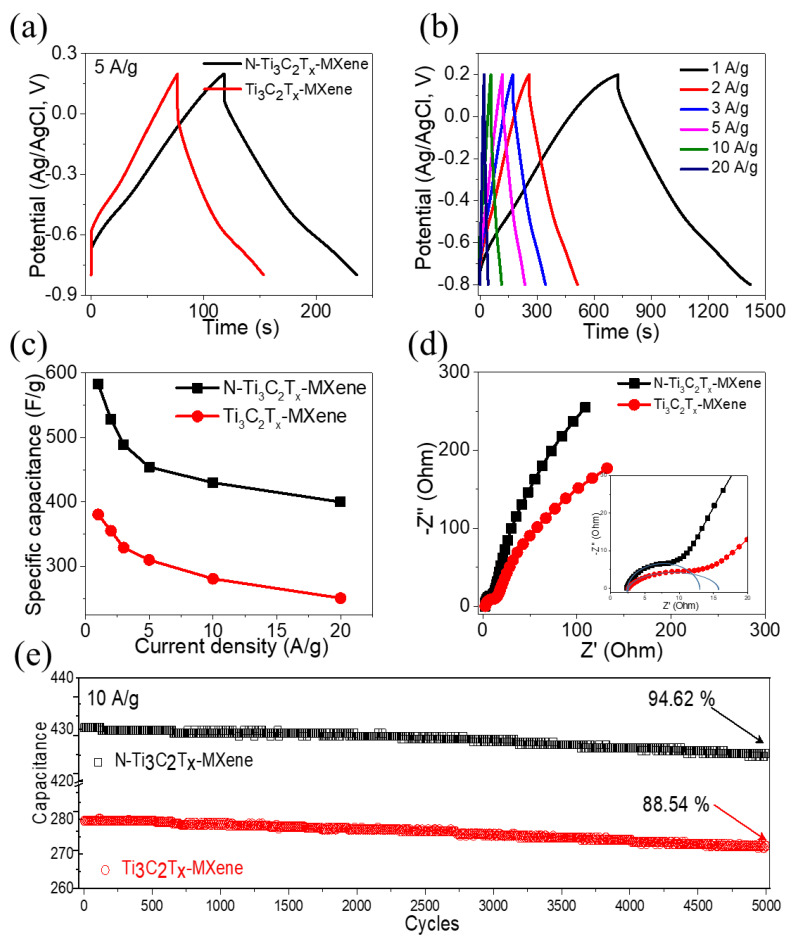
(**a**) GCDs of pristine Ti_3_C_2_T*_x_*-MXene and *N*-Ti_3_C_2_T*_x_*-MXene; (**b**) GCDs of *N*-Ti_3_C_2_T*_x_*-MXene at different current densities; (**c**) specific capacitance of pristine Ti_3_C_2_T*_x_*-MXene and *N*-Ti_3_C_2_T*_x_*-MXene versus current density; (**d**) Nyquist plots; and (**e**) cyclic stability.

Figure 5a displays the GCDs of pristine Ti_3_C_2_T*_x_*-MXene and *N*-Ti_3_C_2_T*_x_*-MXene at 5 A/g. *N*-Ti_3_C_2_T*_x_*-MXene had a charge–discharge duration of 253.35 s, which was significantly longer than the charge–discharge duration of a pristine Ti_3_C_2_T*_x_*-MXene electrode (152.69 s), and was consistent with CV observations. Additionally, Figure 5b displays the *N*-Ti_3_C_2_T*_x_*-MXene electrode’s GCDs at 1 to 20 A/g; excellent capacitive responsiveness with highly reversible charge–discharge at the *N*-Ti_3_C_2_T*_x_*-MXene electrode is indicated by the GCD curves’ symmetry across all current densities. A small IR drop in discharge curves indicates the low internal resistance of the *N*-Ti_3_C_2_T*_x_*-MXene electrode [37]. A longer discharge duration at higher current density values and maintaining symmetry indicate good Coulombic efficiency and outstanding charge storage characteristics [38]. Using discharge times, capacitances were determined according to Equation (1). As seen in Figure 5c, the capacitances of pristine Ti_3_C_2_T*_x_*-MXene and *N*-Ti_3_C_2_T*_x_*-MXene were 582.96 and 380.64 F/g at 1 A/g, respectively. Surprisingly, the *N*-Ti_3_C_2_T*_x_*-MXene retained its capacitance of 400 F/g (68.6%) even at 20 A/g, and it was higher than that of pristine Ti_3_C_2_T*_x_*-MXene (250.66 F/g, 65.8%). The higher capacitance of the *N*-Ti_3_C_2_T*_x_*-MXene electrode was attributed to *N*-functionalization. Figure 5d depicts the Nyquist pattern, which helps explain the increased electrochemical performance of *N*-Ti_3_C_2_T*_x_*-MXene, as determined using EIS tests. The semicircle’s diameter shows a charge transfer resistance in the high-frequency zone of R_ct_ ~ 10.71 Ω for the *N*-Ti_3_C_2_T*_x_*-MXene electrode, which was lower than that of pristine Ti_3_C_2_T*_x_*-MXene (R_ct_ ~ 13.3 Ω). The computed equivalent series resistance R_s_ from the x-intercept for *N*-Ti_3_C_2_T*_x_*-MXene was R_s_ ~ 2.39 Ω, whereas it was R_s_ ~ 2.56 Ω for the Ti_3_C_2_T*_x_*-MXene electrode. This demonstrates *N*-Ti_3_C_2_T*_x_*-MXene’s higher electrical conductivity due to *N*-functionalization. The cyclic life of pristine Ti_3_C_2_T*_x_*-MXene and *N*-Ti_3_C_2_T*_x_*-MXene were evaluated at 10 A/g. The cyclic stability of pristine Ti_3_C_2_T*_x_*-MXene and *N*-Ti_3_C_2_T*_x_*-MXene electrodes are depicted in Figure 5e. The *N*-Ti_3_C_2_T*_x_*-MXene electrode exhibited 99.62% retention for 5000 cycles, whereas pristine Ti_3_C_2_T*_x_*-MXene demonstrated 88.54% retention for 5000 cycles; the *N*-Ti_3_C_2_T*_x_*-MXene electrode had exceptional cyclic performance. 

**Figure 6:**
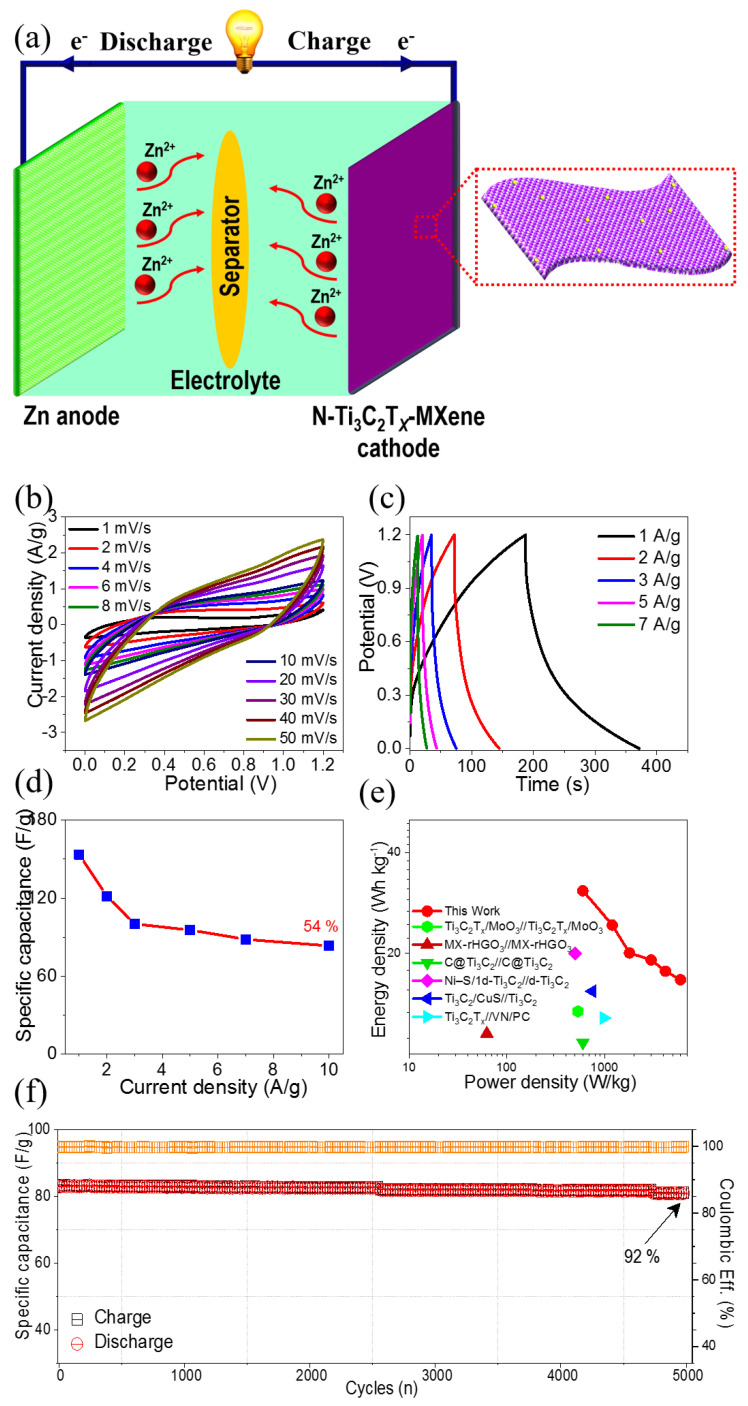
Electrochemical characterization of *N*-Ti_3_C_2_T*_x_*-MXene//Zn in a two-electrode setup: (**a**) schematic illustration of *N*-Ti_3_C_2_T*_x_*-MXene//Zn device; (**b**) CVs of *N*-Ti_3_C_2_T*_x_*-MXene//Zn in 0.0 to 1. 2 V; (**c**) GCDs at 1 to 7 A/g; (**d**) capacitance versus current density; (**e**) Ragone plot; and (**f**) cyclic stability.

In conjunction with their potential window, the improved energy storage performance of *N*-Ti_3_C_2_T*_x_*-MXene electrodes in a three-electrode system suggested that a two-electrode energy storage device constructed from such material might exhibit excellent performance. Therefore, to investigate the viability of the *N*-Ti3C2Tx-MXene electrode for use in real-world applications, an aqueous *N*-Ti_3_C_2_T*_x_*-MXene//Zn device was assembled. Figure 6a illustrates the assembly process and operation of an aqueous *N*-Ti_3_C_2_T*_x_*-MXene//Zn device using a 1 M ZnSO_4_. Figure 6b displays CV curves for the *N*-Ti_3_C_2_T*_x_*-MXene//Zn device using a potential window of 0.0 to 1.2 V, where preservation of CVs’ shapes demonstrates excellent electrochemical stability. Further, the area under CV curves gradually increased with sweep rates, indicating that the *N*-Ti_3_C_2_T*_x_*-MXene//Zn device had superior electrochemical performance. In addition, GCD measurements were performed from 1 to 7 A/g, as shown in Figure 6c. GCD curves for the *N*-Ti_3_C_2_T*_x_*-MXene//Zn device demonstrate both highly reversible charge–discharge curves and high Coulombic efficiency. The maximum capacitance (C_d_) of the *N*-Ti_3_C_2_T*_x_*-MXene//Zn device was calculated according to Equation (2). The maximum computed specific capacitance was 153.55 F/g at 1 A/g, as illustrated in Figure 6d. At 10 A/g, 54% capacitance was retained, which indicated the *N*-Ti_3_C_2_T*_x_*-MXene//Zn device’s exceptional rate performance. Figure 6e displays the Ragone plot, which demonstrates the E and P of the *N*-Ti_3_C_2_T*_x_*-MXene//Zn device (calculated according to Equations (3) and (4), respectively). The *N*-Ti_3_C_2_T*_x_*-MXene//Zn device delivered energy densities of 30.7, 24.3, 20.1, 19.1, 17.7, and 16.7 Wh/kg at power densities of 600.5, 1200.9, 1801.4, 3002.4, 4203.4, and 6004.8 W/kg, respectively. This indicates its superiority compared to most previously explored symmetric/asymmetric SCs devices [35,39,40,41,42,43]. Furthermore, Figure 6f displays the cyclic stability of the *N*-Ti_3_C_2_T*_x_*-MXene//Zn device, which exhibited 92% retention for 5000 cycles, indicating exceptional cyclic stability with nearly 100% Coulombic efficiency. According to the findings of the single electrode and ZISCs device, *N*-Ti_3_C_2_T*_x_*-MXene material demonstrates outstanding electrochemical performance as indicated by specific capacitance, cyclic performance, energy density, and power density. We believe that these exceptional properties are due to the favorable *N*-functionalization of the *N*-Ti_3_C_2_T*_x_*-MXene electrode material. In particular, *N*-atoms’ presence enabled *N*-Ti_3_C_2_T*_x_*-MXene to provide a relatively high active surface area and excellent electrochemical activity. 

## 4. Conclusions

In conclusion, we presented a straightforward fabrication method for a newly designed *N*-Ti_3_C_2_T*_x_*-MXene via a hydrothermal reaction with urea. *N*-functionalization into Ti_3_C_2_T*_x_*-MXene raised the interlayer spacing of Ti_3_C_2_T*_x_*-MXene. The fabricated *N*-Ti_3_C_2_T*_x_*-MXene delivered a maximum capacitance of 582.96 F/g at 1 A/g and retained 94.62% capacitance for 5000 cycles at 10 A/g. Additionally, the *N*-Ti_3_C_2_T*_x_*-MXene//Zn device demonstrated a maximum capacitance of 153.55 F/g at 1 A/g, with 92% retained capacitance for 5000 cycles and a Coulombic efficiency of ~100%. The *N*-Ti_3_C_2_T*_x_*-MXene//Zn device revealed an energy density of 30.7 Wh/kg with a power density of 600.5 W/kg. This research provided insight into the rational assembling of innovative electrode materials for ZISCs.

## Data Availability

Not applicable.
